# High Prevalence of Preexisting HBV Polymerase Mutations in Pregnant Women Does Not Limit the Antiviral Therapy Efficacy

**DOI:** 10.1155/2021/6653546

**Published:** 2021-04-19

**Authors:** Jing Wang, Jinfeng Liu, Qiang Yu, Li Jin, Naijuan Yao, Yuan Yang, Taotao Yan, Chunhua Hu, Yingli He, Yingren Zhao, Tianyan Chen, Jie Zheng

**Affiliations:** ^1^Department of Rheumatology, First Affiliated Hospital, School of Medicine, Xi'an Jiaotong University, Xi'an, Shaanxi 710061, China; ^2^Department of Infectious Diseases, First Affiliated Hospital, School of Medicine, Xi'an Jiaotong University, Xi'an, Shaanxi 710061, China; ^3^Department of Pediatric Surgery, Second Affiliated Hospital, School of Medicine, Xi'an Jiaotong University, Xi'an, Shaanxi 710061, China; ^4^Department of Nephrology, First Affiliated Hospital, School of Medicine, Xi'an Jiaotong University, Xi'an, Shaanxi 710061, China; ^5^Clinical Research Center, First Affiliated Hospital, School of Medicine, Xi'an Jiaotong University, Xi'an, Shaanxi 710061, China

## Abstract

**Background:**

HBV-resistant mutants in treatment-naïve patients may lead to antiviral treatment failure. It is not clear if HBV mutants are present in pregnant women and about the influence of the preexisting mutants on the short-term antiviral therapy during pregnancy.

**Method:**

We enrolled 73 pregnant women with high HBV DNA load and telbivudine (TBV) treatment during pregnancy in this retrospective study. The UDPS was used to detect the HBV mutations before and after the TBV treatment.

**Results:**

Before TBV treatment, the complexity of HBV quasispecies of all subjects was 0.40 ± 0.09; 41.1% (30/73) and 53.4% (39/73) subjects had rtM204I/V and rtN236 T/A detected, respectively; and 9.6% (7/73) patients had more than 20% frequency mutation of rtM204I/V, which was also similar with high frequency of rtN236 T/A mutation (41.1% vs. 53.4%, *P*=0.136; frequencies >20%: 9.6% vs. 5.5%, *P*=0.347). After TBV treatment, 71.2% (52/73) subjects had HBV DNA load ≥ 10^3^ IU/mL at delivery. Among them, 75.0% of patients with rtM204I positive had HBV DNA load ≥10^3^ IU/mL at delivery, which was comparable with the subjects without rtM204I (75.0% vs. 70.8%, *P*=0.710). No changes were found in the frequencies and the complexity of HBV quasispecies of rtM204I mutation after the TVB treatment.

**Conclusion:**

The prevalence of preexisting drug-resistant mutations among pregnant women was high using UPDS. However, the preexisting HBV mutation had limited influence on the efficacy of short-term TBV treatment, and TBV treatment during late pregnancy seemed not to increase the risk of emerging HBV-resistant mutants.

## 1. Introduction

The current guidelines recommend that pregnant women with high HBV DNA levels should accept antiviral prophylaxis in gestation [1–3]. It was recommended for pregnant women to decrease the HBV DNA load below a relatively safe threshold for the prevention of HBV mother-to-infant transmission (MTIT) during the third trimester [4].

The nucleoside/nucleotide analogues (NAs) are able to suppress HBV replication by inhibiting the viral reverse transcriptase (RT); however, HBV RT has no proofreading activity. It increases the HBV mutations and promotes genetic diversity, which may cause drug resistance [5, 6]. Studies showed that some resistance mutations related to NAs therapy might already be present in treatment-naïve patients [7–9]. It is reported that YMDD mutations were present in a subgroup of NA-naïve patients with a frequency ranging from 3% to 27% [10–13]. HBV-resistant mutants in treatment-naïve patients may lead to drug resistance and treatment failure [14]. It is not clear if HBV-resistant mutants are present in pregnant women before the antiviral treatment.

Tenofovir (TDF) and Telbivudine (TBV) classified as category B were the NAs recommended by several clinical practice guidelines and widely used to prevent MTIT. TDF was considered with a high genetic barrier to HBV resistance. Similar to lamivudine (LAM), TBV was with a low genetic barrier to HBV resistance. It was observed for LAM, a drug-resistant viral variant, among mothers with high HBV load who received LAM treatment from 22 to 88 days during the pregnancy [15], while the study of TBV is still limited. Yingxia Liu et al. reported that one of the 50 high HBV DNA loads subjects developed rtM204I drug-resistance mutation after receiving TBV treatment, but the time duration that the patient received TBV treatment in the study was not clear [16]. Another prospective study did not find the rtM204 mutations among the participants who started TBV 600 mg/day at week 20 to week 32 of gestation and stopped TBV one month postpartum [17]. The clinical impact of short-duration TBV usage should be studied further in high-risk pregnant women.

The objective of this study is to assess the prevalence of HBV preexisting resistant mutants in pregnant women and explore the influence of the preexisting resistant mutants on the efficacy of short-term TBV therapy during pregnancy. We used ultradeep pyrosequencing (UDPS) to sequence HBV and detect low-level (<1.0%) clinically relevant variants within complex viral populations.

## 2. Materials and Methods

### 2.1. Participants

This was a retrospective study; all data were collected from another cohort study [18]. 73 chronic HBV infected pregnant women with high HBV DNA load undergoing routinely consultation from March 1^st^, 2012, to May 31^st^, 2015, were recruited from the First Affiliated Hospital of Xi'an Jiaotong University, Shaanxi, China. Inclusion criteria included pregnant women aged from 18 to 40 years, who had serum HBsAg positive for more than 6 months and HBV DNA load greater than 10^6^ IU/ml, and who started taking TBV (600 mg/day) from the 24^th^ week of gestation and stopped TBV 12 weeks postpartum. Exclusion criteria were if patients were serologic HIV or hepatitis C or hepatitis *D* virus-positive or if patients had anti-HBV treatment before the 24^th^ week of gestation during the pregnancy, took the immunosuppressive agents during the pregnancy, and were diagnosed as any of the following diseases: gestational diabetes, arrhythmia, anemia, or proteinuria. All patients were evaluated every 4 weeks from the 24^th^ week of gestation, at delivery, and at postpartum weeks (PPW) 4, 12, 24, and 52. The study was approved by the ethics committee of the First Affiliated Hospital of Xi'an Jiaotong University. Informed consent was obtained from each participant.

All infants born to the chronic HBV infected mothers received combined immunoprophylaxis, 200 IU of hepatitis B immunoglobulin, and 10 *μ*g of recombinant HBV vaccine within 12 h postpartum, at 1 month, and at 6 months.

### 2.2. Ultradeep Pyrosequencing Data

To evaluate the risk of HBV drug resistance generated by the short duration of TBV in pregnancy, polymerase gene analysis was conducted by using UDPS prior to (at the 24^th^ week of gestation) and after (at the last time point of follow-up) TBV treatment. The HBV RT was ampliﬁed (697 bp) with the primers Seq2 (5′-TTGGCCAAAATTCGCAGTC-3′) and OS2 (5′-TCTCTGACATACTTTCCAAT-3′) [15]. The PCR products were purified using an Omega gel extraction kit (Omega Bio-Tek, USA) and quantified by a Nanodrop 1000 (Thermo Scientific, Wilmington, USA). UDPS was performed on the 454 Life Science platform (GS FLX platform, Roche).

The sensitivity of UDPS on the 454 Life Science platform for detecting low-level viral variants at 0.1% to 1% has been confirmed by the use of standard cloning methods [19–21]; the variants with prevalence larger than 1% were classified as high-confidence variants.

The UDPS generated sequence reads were filtered using the following criteria: (1) mismatched base number of 5′ primers greater than 1, (2) no undetermined bases, (3) continuous same bases greater than 8, (4) 150 bases in length or less, and (5) chimera sequence. The average number of reads generated for each sample was 9766 (range: 1913 to 21909). The filtered sequence reads were aligned to their respective consensus sequences, the Smith–Waterman algorithm and mutations in corresponding sites were used to calculate Sanger sequences.

### 2.3. HBV Quasispecies Complexity of AA

The HBV quasispecies complexity of AA level was estimated for each site using Shannon entropy (Sn) [22, 23], which can be calculated with the formula Sn = −∑_*i*_(*p*_*i*_Ln*p*_*i*_)/Ln*N*, where *N* is the total number of clones and *p*_*i*_ is the frequency of each clone in the viral quasispecies population [24]. The mean viral complexity in each sample was calculated by the ratio of total amounts of the Sn at each position and the total length AA number. Mutations of rtL80, rtL82, rtV84, rtS85, rtI91, rtI169, rtV173, rtL180, rtA181, rtT184, rtA194, rtA200, rtS202, rtM204, rtV207, rtS213, rtV214, rtQ215, rtL217, rtE218, rtF221, rtL229, rtI233, rtN236, rtP237, rtN/H238, rtY245, rtM250, and rt S/C256 were analyzed in this study.

### 2.4. Other Measurements

Data of age, parity, antiviral treatment history before pregnancy, HBV family history, patients HBVDNA load, HBV serum markers titer including HBsAg and HBeAg, alanine transaminase (ALT) level, and creatinine kinase (CK) at 24^th^, 28^th^, 32^th^, and 36^th^ weeks of gestation, delivery, postpartum weeks (PPW) 4, 12, 24, and 52, and corresponding safety data of infants were collected from the medical records of the hospital.

### 2.5. Statistical Analysis

Continuous variables were presented as means ± standard deviations and categorical variables were presented as counts (percentages). Paired *t*-tests were used to test the changes of the complex of HBV quasispecies before and after TBV treatment. The frequency of the mutations at rtM204 was compared using *t*-tests between patients with and without plasma HBV DNA<10^3^ IU/mL at delivery. All tests were two-side tests, and *P* values < 0.05 were considered statistically significant. All analyses were performed with SPSS software 24.0 (SPSS Inc., Chicago, IL, USA).

## 3. Results

### 3.1. Baseline Maternal Characteristics

Total 73 HBsAg (+) and HBV DNA load > 10^6^ IU/mL pregnant women were enrolled in the current study. Subjects accepted TBV from the 24^th^ week of gestation to PPW 12 and then were followed up at least to PPW 52. The median follow-up time was 76 weeks (range: 52–152 weeks). The baseline demographics and clinical characteristics of the mothers are summarized in Table 1. Six pregnant women (8.2%) accepted antiviral treatment before pregnancy, 3 had interferon treatment, and 3 had LAM treatment.

### 3.2. Dynamics of Maternal HBV DNA Load

**Figure 1:**
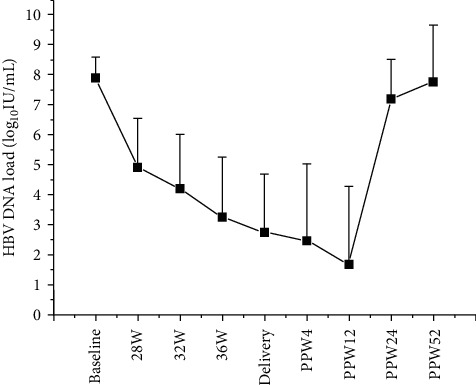
HBV DNA load kinetics in pregnancy and postpartum. HBV: hepatitis B virus; W: week; PPW: postpartum week.

As shown in Figure 1, compared to baseline, TBV treatment reduced HBV DNA level (4.36 ± 2.03, range 1.84 to 8.95 log_10_ IU/mL) in all mothers. There were 52 out of 73 (71.2%) women who had serum HBV DNA load more than 10^3^ IU/mL at delivery. Viral breakthrough was not observed during TBV treatment. After TBV withdrawal, HBV DNA levels rebounded in all mothers and reached a mean of 7.21 ± 1.34 log_10_ IU/mL after 3 months of the withdrawal (PPW 24).

### 3.3. Viral Quasispecies Complexity and NA-Resistant Mutations before TBV Treatment

**Figure 2:**
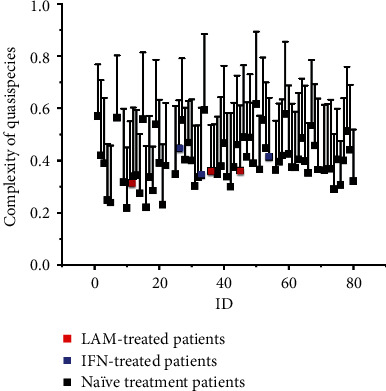
Scatter diagram of HBV quasispecies complexity. IFN: interferon; LAM: lamivudine.

The complexity of viral quasispecies of the samples was calculated as described in Methods section. The complexity of quasispecies (*Sn*) before TBV treatment in all patients was 0.40 ± 0.09. The Sn value of treatment-naïve patients was 0.40 ± 0.10. The Sn values of the three patients who accepted LAM before pregnancy were 0.31, 0.36, and 0.36, respectively. The Sn values of the three patients who accepted interferon treatment before pregnancy were 0.45, 0.34, and 0.41, respectively (Figure 2).

The 29 known NAs-resistant mutations [25, 26] were analyzed in the 73 pregnant women. At primary drug resistance mutation sites, rtM204I/V associated with resistance to LAM and TBV, also known as classical YMDD mutation, presented in 41.1% (30/73) of patients before TBV treatment and 9.6% of patients had mutation frequencies greater than 20%; RtA181 T/V was in 5.5% (4/73) of patients, involved in the LAM, TBV, and ADV shared resistance pathway; and rtN236 T/A mutation, which was reported to decrease the sensitivity to TDF, presented in 53.4% (39/73) pregnant women and 5.5% of patients had mutation frequencies greater than 20%. The proportions of patients with rtN236 T/A mutation have no difference with those of patients with rtM204I/V mutation (41.1% vs. 53.4%, *P*=0.136; frequencies greater than 20%: 9.6% vs. 5.5%, *P*=0.347). At the compensatory mutation sites, 15.1% (11/73) participants had L80I/V mutation that is associated with resistance to LAM. In addition, the patients prior to TBV treatment also had other putative antiviral resistance mutations (Table 2). However, rtI169 T, rtA194 T, rtV173 L, rtL180 M, rtL82 M, rtS85 A, rtV207I, rtL217 R, and rtS/C256G mutations were not present before TBV treatment. Two patients who accepted LAM before pregnancy had preexisting rtM204I mutation.

As shown in Table 3, 34.3% (25/73) patients had rtM204I mutation and 27.4% (20/73) had rtM204 V mutation. Multibase mutations combined with rtM204I/V were analyzed; rtM204I + rtN236 T and rtM204 V + rtN236 T appeared to be the most common ones (16.4% and 17.8%, resp.). RtM204I/V + rtL80I/V and rtM204I + rtA181 T/V may affect the sensitivity to LAM, TBV, and ADV; they also presented but the proportions of the mutations were low (Table 3).

### 3.4. Preexisting HBV Mutations and the TBV Treatment Effect

**Figure 3:**
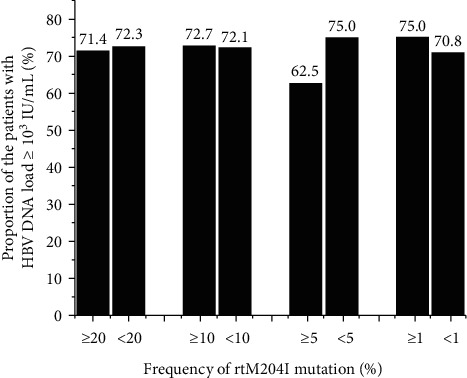
Relation between the frequency of rtM204I and maternal HBV DNA load at delivery. HBV: hepatitis B virus.

After receiving TBV treatment during pregnancy, 71.2% (52/73) of patients still had HBV DNA load ≥ 10^3^ IU/mL at delivery. The complex of HBV quasispecies of these patients was not found to be significantly different from that of the patients with HBV DNA load less than 10^3^ IU/mL at delivery (0.40 ± 0.09 vs. 0.40 ± 0.09, *P*=0.353). The frequency of rtM204I was not significantly higher in patients with HBV DNA load ≥10^3^ IU/mL than that of patients with HBV DNA <10^3^ IU/mL at delivery (0.13 ± 0.12 vs. 0.15 ± 0.11, *P*=0.669), either. Among them, 75.0% of patients with rtM204I positive had HBV DNA load ≥10^3^ IU/mL at delivery, which was comparable with the subjects without rtM204I (75.0% vs. 70.8%, *P*=0.710). In addition, the patients were further divided into high mutation group (the frequency of rtM204I ≥ 20%, 10%, and 5%) and low mutation group (the frequency < 20%, 10%, and 5%) before the TBV treatment. As shown in Figure 3, the proportion of maternal HBV DNA load ≥10^3^ IU/mL at delivery was 71.4% in the rtM204I ≥ 20% group, which was similar to that in the rtM204I < 20% group (71.4% vs. 72.3%, *P*=0.961). Similar trend was observed in groups with 10% (72.7% vs. 72.1%, *P*=0.968) and 5% frequency of rtM204I (62.5% vs. 75.0%, *P*=0.325).

### 3.5. HBV Mutations after the Short-Term TBV Treatment

**Figure 4:**
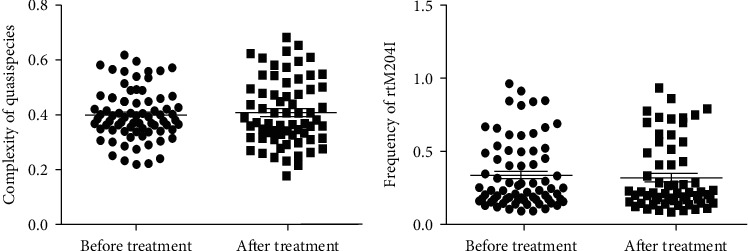
Impact of telbivudine short-time treatment to HBV mutations: (a) the change in HBV quasispecies complexity before and after telbivudine treatment; (b) the change in rtM204I mutation frequency before and after telbivudine treatment.

The impact of TBV short-time treatment on HBV mutations was analyzed among the 73 pregnant women. No change was found in the frequencies of rtM204I mutation before and after (0.34 ± 0.23 vs. 0.32 ± 0.23, *P*=0.681, Figure 4(b)) the TVB treatment. Compared with the HBV quasispecies complexity at baseline, there was no significant increase after the TBV treatment (0.40 ± 0.09 vs. 0.41 ± 0.12, *P*=0.599, Figure 4(a)) as well.

### 3.6. Safety of TBV Treatment

TBV treatment was generally tolerated well by the mothers and their infants; there were no maternal severe adverse effects observed in this study. Mild creatinine kinase (CK) elevation (<2 × ULN) was reported for 1 of 73 mothers (1.4%), and CK level normalized after telbivudine withdrawal (Table S1). Among the 73 infants, there was no preterm, low birth weight, and Apgar scores <10 infants, and none of them had congenital deformities. No infant was found seropositive for HBsAg, HBeAg, and HBV DNA in the follow-up.

## 4. Discussion

TBV was an antiviral agent with a low genetic barrier to HBV resistance. The efficacy of the TBV treatment was easily limited in the patients with some HBV resistance mutations, and the new resistance mutations were easily developed by TBV treatment. In our study, TBV, a low resistance barrier agent, was administrated in the pregnant women and the HBV RT sequences were tested before and after TBV treatment; we found that the overall viral quasispecies complexity was 0.40 ± 0.09 and 30 of 73 patients (41.1%) had rtM204I/V positive at baseline, while 71.2% of pregnant women had serum HBV DNA load more than 10^3^ IU/mL at delivery.

It was reported that the preexisting primary resistance mutations could reduce the susceptibility of anti-HBV monotherapy or even combined-therapy; for example, rtM204I was refractory to LAM and TBV, and the efficacy of the corresponding NAs could be affected [26]. High frequency of rtM204I mutation (more than 20%) can be tested in approximately 30% of patients after 104 weeks of TBV treatment, and virological breakthrough was observed as well [27]. To date, there is no study directly focusing on the association between preexisting resistance mutations and the short-term antiviral treatment efficacy. We tested the association between the mutation frequency of rtM204I and the HBV DNA load decrease; no significant association was found in either the high mutation frequency group or low mutation frequency group (Figure 3). Moreover, mutation frequency more than 30% was also analyzed; the pregnant women with higher mutation frequency did not have more proportion of HBV DNA load ≥10^3^ IU/mL at delivery compared with those patients with rtM204I < 30% (50.0% vs. 72.9%, *P*=0.481). It indicated that the preexisting primary resistance might have no influence on the short-term TBV treatment. We analyzed other mutations in the same way; no mutations were found to be associated with the HBV DNA load decline as well (data were not shown). Besides, rtN236 T/A mutation that was related to decreasing sensitivity of TDF presented in 53.4% of the pregnant women, which had no difference with the proportion of the patients with rtM204I/V mutation. This indicated that TDF with a high genetic barrier to HBV resistance might not be superior to TBV for pregnant women to prevent MTIT from the view of preexisting primary resistance mutations.

We used UDPS to detect the drug resistance mutations, which is much more sensitive than the methods of many studies used before (5% to 20% variants detected in NAs-naïve patients) [10–12]. The UDPS can detect minor HBV variants and reveal the massive genetic heterogeneity by parallel ampliﬁcation and detection of abundant small size sequences [28]; moreover, it can provide longer reads than other techniques and is suitable for viral resistance studies [29]. As far as we know, this is the first work to evaluate the preexisting NA resistance mutations by UDPS in a moderate sample of pregnant women with chronic HBV infection. In the present study, 41.1% of patients were rtM204I/V positive, while only 9.6% (7/73) patients had rtM204I/V frequency of 20% or more. In addition, the average frequency of the mutation was 0.13 ± 0.11, both of which were consistent with the previous study findings [10–12]. Two previous studies conducted rtM204I/V mutation testing with sensitive methods. Kirishima et al. reported 22.2% (4/18) NA-naïve patients had rtM204I/V mutation by peptide nucleic acid mediated polymerase chain reaction clamping which could detect mutation rate as low as 0.01–0.001% [13], and Ayres et al. detected 12.5% (3/24) pregnant women had the mutation by UDPS [15], which were lower than the rate in our study; the difference may be associated with the very limited sample sizes in the above two studies.

Drug-resistant HBV variants were reported to emerge in the mothers' accepted short-term LAM treatment during late pregnancy [15]. Han et al. reported rtM204 mutation arose in two mothers at 22 weeks and 71 weeks of TBV treatment, respectively [12]. In our study, approximately 7 months of TBV treatment was administrated in pregnant women. No increases of the viral quasispecies complexity and the frequency of rtM204I mutation were observed, which supplemented the safety of TBV treatment in late pregnancy. Furthermore, some pregnant women had multibase mutations combined with rtM204I/V at baseline, including rtM204I + rtA181 T/V, rtM204I/V + rtL80I/V, rtM204I/V + rtN236 T, and rtM204I/V + rtI233 V, which may affect their sensitivity to LAM, TBV, ADV, and TDF. The complexity of viral quasispecies and the frequency of rtM204I mutation had no significant increase after TBV treatment in those pregnant women; however, caution has to be taken for them to choose NAs in subsequent long-term therapy due to the drug resistance mutations.

In this study, the prevalence of HBV preexisting resistant mutants in pregnant women, the influence of the efficacy of short-term TBV treatment, and the drug-resistant mutations after TBV therapy were assessed retrospectively. Although the number of subjects was moderate, a prospective cohort study with a larger sample size is necessary to evaluate the relationship between the HBV mutations and the short-term antiviral treatment effect.

In conclusion, the prevalence of preexisting HBV mutation among pregnant women was as high as 41.1%. However, the preexisting HBV mutation had limited influence on the efficacy of short-term TBV treatment, and TBV treatment during late pregnancy seemed not to increase the risk of emerging HBV-resistant mutants.

## Figures and Tables

**Table 1:** Demographics and baseline characteristics.

Variable	Value
Age years^*∗*^	27.78 ± 3.89
Parity^*∗*^	1.14 ± 0.35
Previous use of antiviral, number (%)	6 (8.22)
HBV family history, number (%)	32 (43.84)
ALT levels U/L^*∗*^	39.15 ± 43.97
ALT > 40 U/L, number (%)	22 (30.14)
ALT > 80 U/L, number (%)	7 (9.59)
ALT > 200 U/L, number (%)	1 (1.37)
HBV DNA load Log_10_IU/mL^*∗*^	7.91 ± 0.70
HBsAg titer Log_10_IU/mL^*∗*^	4.38 ± 0.47
HBeAg titer Log_10_s/co^*∗*^	2.45 ± 1.26
HBeAg (+), number (%)	63 (86.30)

^*∗*^The values are expressed as means ± standard deviations for continuous variables and number of patients (percentages) for categorical variables. Abbreviations: ALT, alanine transaminase; HBeAg, hepatitis B e antigen; HBsAg, hepatitis B surface antigen; HBV, hepatitis B virus.

**Table 2:** Potential NAs mutation at 29 positions of HBV reverse transcriptase analyzed in the 73 pregnant women.

Mutations type	Relationship with therapy	The proportion of the patients with mutations, *n* (%) (*n* = 73)	The frequency of the mutations^*∗*^ (%)	Patients with mutations frequency>20%, *n* (%)
*Primary resistance mutations*				
rtI169T	ETV	0	0	0
rtA181T/V	LAM, TBV, ADV, TDF	4 (5.5)	0.023 ± 0.020	0
rtT184A/C/F/G/I/L/M/S	ETV	52 (71.2)	0.13 ± 0.14	14 (19.2%)
rtA194T	ADV, TDF	0	0	0
rtS202C/G/I	ETV	1 (1.4)	0.01	0
rtM204I/V	LAM, ETV, TBV	30 (41.1)	0.13 ± 0.11	7 (9.6%)
rtN236T/A	ADV, TDF	39 (53.4)	0.10 ± 0.13	4 (5.5%)
rtM250I/L/V	—	41 (56.2)	0.11 ± 0.08	5 (6.8%)

*Compensatory mutations*				
rtL80I/V	LAM	11 (15.1)	0.02 ± 0.01	—
rtV173L	LAM	0	0	0
rtL180M	LAM, ETV, TBV	0	0	0

*Putative NAs mutations*				
rtL82M	LAM	0	0	0
rtV84M	ADV	2 (2.7)	0.01 ± 0.001	0
rtS85A	ADV	0	0	0
rtI91L	LAM	40 (54.8)	0.82 ± 0.30	36 (49.3%)
rtA200V	LAM	1 (1.4)	0.02	0
rtV207I	LAM	0	0	0
rtS213T	ADV	3 (4.1)	0.11 ± 0.16	1 (1.4%)
rtV214A	ADV	3 (4.1)	0.01 ± 0.004	0
rtQ215P/S	LAM, ADV	12 (16.4)	0.07 ± 0.08	2 (2.7)
rtL217R	ADV	0	0	0
rtE218D	ADV	1 (1.4)	0.52	1 (1.4)
rtF221Y	ADV	30 (41.1)	0.29 ± 0.25	16 (21.9)
rtL229G/V/W	LAM	24 (32.9)	0.05 ± 0.06	1 (1.4)
rtI233V	ADV	30 (41.1)	0.12 ± 0.11	7 (9.6)
rtP237H	ADV	22 (30.1)	0.02 ± 0.02	0
rtN/H238D/S/T/A	ADV	46 (63.0)	0.15 ± 0.12	7 (9.6)
rtY245H	ADV	1 (1.4)	0.13	0
rtS/C256G	LAM, ETV	0	0	0

^*∗*^The prevalence of mutations were expressed as number of patients (percentages) and the mutation frequencies were expressed as mean ± standard deviation. Abbreviations: ADV, adefovir dipivoxil; ETV, entecavir; HBV, hepatitis B virus; LAM, lamivudine; n, number; NA, nucleoside/nucleotide analogues; TBV, telbivudine; TDF, tenofovir disoproxil fumarate.

**Table 3:** The multi-base mutations combined with rtM204I/V.

Types of mutation patterns	The rate of the patients with mutations, n (%)
	(*n* = 73)
*rtM204I*	
rtM204I alone	25 (34.3)
rtM204I + rtL80I/V	5 (6.8)
rtM204I + rtA181T/V	2 (2.7)
rtM204I + rtL180M	0
rtM204I + rtN236T	12 (16.4)
rtM204I + rtI233V	12 (16.4)
rtM204I + rtA194T	0

*rtM204V*	
rtM204V alone	20 (27.4)
rtM204V + rtL80I/V	5 (6.8)
rtM204V + rtA181T/V	0
rtM204V + rtL180M	0
rtM204V + rtN236T	13 (17.8)
rtM204V + rtI233V	9 (12.3)
rtM204V + rtA194T	0

Abbreviations: *n*, number.

## Data Availability

The data in the current study are available from the corresponding author on reasonable request.

## Supplementary Materials

Supplementary Materials Table S1: adverse events reported in the current study*∗*.
