# Supplementary material for: High Prevalence of Preexisting HBV Polymerase Mutations in Pregnant Women Does Not Limit the Antiviral Therapy Efficacy
Source: Can J Infect Dis Med Microbiol. 2021 Apr 19;2021:6653546. doi: 10.1155/2021/6653546 (PMC8079218; doi:10.1155/2021/6653546)
Supplement: Supplementary Materials — Table S1: adverse events reported in the current study∗. [file 6653546.f1.docx]

**Table S1.** **Adverse events reported in current study***

| **Adverse event** | **Number (%)** |
| --- | --- |
| Gastrointestinal symptom | 5 (6.85%) |
| Nausea | 1 (1.37) |
| Emesis | 1 (1.37) |
| Diarrhea | 1 (1.37) |
| flatulence | 1 (1.37) |
| Dyspepsia | 1 (1.37) |
| Fatigue | 2 (2.74) |
| Dizziness | 1 (1.37) |
| Insomnia | 2 (2.74) |
| Myalgia | 1 (1.37) |
| CK elevation | 1 (1.37) |
| Cesarean delivery | 35 (47.95) |
| Threatened abortion | 11 (15.07) |
| Prolonged Labor | 4 (6.25) |

*Number (percentages) of patients with adverse events were reported. Abbreviations: CK, creatinine kinase.
